# The Role of ^68^Ga-PSMA Positron Emission Tomography/Computerized Tomography for Preoperative Lymph Node Staging in Intermediate/High Risk Patients With Prostate Cancer: A Diagnostic Meta-Analysis

**DOI:** 10.3389/fonc.2020.01365

**Published:** 2020-08-18

**Authors:** Xiang Tu, Chichen Zhang, Zhenhua Liu, Guohua Shen, Xiaoai Wu, Ling Nie, Tiancong Chang, He Xu, Yige Bao, Lu Yang, Qiang Wei

**Affiliations:** ^1^Department of Urology, Institute of Urology, West China Hospital, Sichuan University, Chengdu, China; ^2^West China School of Clinical Medicine, West China Hospital, Sichuan University, Chengdu, China; ^3^Laboratory of Clinical Nuclear Medicine, Department of Nuclear Medicine, West China Hospital, Sichuan University, Chengdu, China; ^4^State Key Laboratory of Biotherapy, Department of Pathology and Laboratory of Pathology, West China Hospital, Sichuan University, Chengdu, China

**Keywords:** lymph node dissection, meta-analysis, positron emission tomography, PET/CT, prostate cancer, prostate-specific membrane antigen

## Abstract

**Purpose:** To evaluate the accuracy of ^68^Ga-PSMA positron emission tomography/computerized tomography (PET/CT) for preoperative lymph node staging using histopathological results of pelvic lymph node dissection (PLND) as reference standard in patients with intermediate/high risk of prostate cancer.

**Material and Methods:** A systematic search of PubMed, Embase, and the Cochrane Library was completed up to May 2020. We included studies investigating accuracy of ^68^Ga-PSMA PET/CT in primary lymph node staging before radical prostatectomy and PLND. The pooled sensitivity, specificity, positive predictive value (PPV), negative predictive value (NPV), diagnostic odds ratio (DOR), and the summary receiver operating characteristic (SROC) curve with an area under the curve (AUC) were synthesized.

**Results:** Eleven studies comprising 904 patients were identified. Based on per-patient analysis, the pooled sensitivity and specificity reached 0.63 (95% CI: 0.46–0.78) and 0.93 (95% CI: 0.88–0.96), respectively, with the DOR of 22 (95% CI: 10–47). An overall accuracy was revealed by the SROC curve with AUC of 0.91 (95% CI: 0.88–0.93). Using the lymph node as unit, the pooled sensitivity and specificity were 0.70 (95% CI: 0.49–0.85) and 0.99 (95% CI: 0.96–1.00), respectively. And the DOR reached 167 (95% CI: 40–695) with an AUC of 0.96 (95% CI: 0.94–0.98). The pooled PPV and NPV all reached above 0.8 on basis of per-patient or per-node analysis.

**Conclusions:** ^68^Ga-PSMA PET/CT represented as a promising test for preoperative lymph node staging and patients without lymph node metastatic status can rarely be misdiagnosed. However, its sensitivity ought to be improved before forgoing PLND.

## Introduction

Prostate cancer (PCa) is regarded as the most commonly diagnosed cancer in men in western countries (1). Radical prostatectomy (RP) remains one widely used curative treatment for patients with localized PCa (2). However, nearly 15% of patients, especially those of intermediate and high risk, harbor lymph node invasion (LVI) during RP with extended pelvic lymph node dissection (PLND) (3). Accurate staging of lymph nodes is important which helps determine the optimal multimodal treatment to achieve better cancer control for patients with metastatic nodes (4).

Currently, PLND remains the gold standard for lymph node staging which provides important information for prognosis (5). However, the frequent complications (lymphocele/lymphedema/venous thromboembolism), high cost, as well as lack of evidence to improve oncological outcomes limited the routine application of this invasive procedure in clinical practice (6, 7). Multiple preoperative nomogram tools have been identified to figure out optimal candidates for PLND, however, lacking an ideal cut-off value because of various patient selections (8–10). In addition, such models were originated from general populations which may not be perfect for one specific individual (11). More specific and non-invasive imaging modalities including computed tomography (CT) and magnetic resonance imaging (MRI) have been assessed for lymph node staging but without satisfactory accuracy (12, 13).

Prostate-specific membrane antigen (PSMA) is overexpressed on the surface of most PCa cells (14). And ^68^Ga-PSMA ligand positron emission tomography/computerized tomography (PET/CT) has been reported to be superior to conventional modalities in the detection of metastatic PCa, especially in the identification of recurrent PCa after primary treatment failure (15, 16). However, the diagnostic role of ^68^Ga-PSMA PET/CT in primary lymph node staging before RP has yet to be fully revealed. We conducted this meta-analysis and tried to evaluate the accuracy of ^68^Ga-PSMA PET/CT for preoperative lymph node staging using histopathological results of dissected lymph nodes as reference standard in patients with intermediate/high risk of PCa.

## Materials and Methods

### Search Strategy

A systematic review was performed in accordance with Preferred Reporting Items for Systematic Review and Meta-analysis (PRISMA) guidelines (17). PubMed, Embase, and the Cochrane Library were searched up to May 2020. The search strategy was applied to identify all trials by using medical subject headings in combination with keywords of “Positron-Emission Tomography,” “PSMA,” “Gallium,” and “prostate cancer.” We limited studies to human. We also manually screened the references of included studies for additional citations.

### Selection Criteria

Two authors evaluated all potential articles independently. The inclusion criteria were as follows: (1) studies investigating the accuracy of ^68^Ga-PSMA PET/CT in primary lymph node staging; (2) patients underwent preoperative ^68^Ga-PSMA PET/CT imaging test before RP with PLND; (3) the patients recruited in the studies did not receive other treatment (hormone treatment, pelvic radiation, or chemotherapy) for PCa previously and had no previous/concurrent other malignancies; (4) relevant data in terms of ^68^Ga-PSMA PET/CT and histopathological results (positive/negative) for PLND can be extracted. We excluded studies which evaluated the utility of the ^68^Ga-PSMA PET/CT in the secondary staging for patients (detecting recurrent PCa after primary treatment failure). We also excluded studies of case reports or series (<10 patients), conference abstracts, and reviews.

### Data Extraction

We extracted basic characteristics including study design, recruiting place and time, and study inclusion criteria. Participant details included age and prostate specific antigen (PSA). PET/CT test details (CT technique, uptake time, definition of positive imaging test) and details of reference standard were also summarized. Outcome data in terms of ^68^Ga-PSMA PET/CT and pathological results (positive/negative) for PLND on the basis of patient unit (per-patient) or node unit (per-node) were both extracted into 2 × 2 contingency tables.

### Quality Assessment

We set ^68^Ga-PSMA PET/CT imaging prior to RP and PLND as the index test. And the histopathological status of the lymph nodes after PLND was defined as the reference standard. The Quality Assessment Tool for Diagnosis Accuracy Studies (QUADAS-2) was used to evaluate the quality of each trial, which included four domains: patient selection, index test, reference standard, and participant flow and timing (18). We judged a study to have “low risk of bias” if it was evaluated as “low” on all four domains. A study might be evaluated as a high risk of bias if more than one domain (including one) was judged “high” or “unclear.” Any discrepancies were resolved by a third reviewer.

### Statistical Analysis

The pooled sensitivity, specificity, positive predictive value (PPV), negative predictive value (NPV), and the summary receiver operating characteristic (SROC) curve with an area under the curve (AUC) of ^68^Ga-PSMA PET/CT test were synthesized. Positive likelihood ratio (PLR), negative likelihood ratio (NLR), and diagnostic odds ratio (DOR) were also pooled to better evaluate diagnostic role of the index test. The results were based on a per-patient analysis (setting one patient as unit) and a per-node analysis (setting one node as unit). More specifically, per-patient analysis (or per-node analysis) was calculated by the proportions of patients (or lymph nodes) with positive/negative histological results who (or which) were correctly identified by PSMA PET/CT. Heterogeneity was valued with the Chi-square and the Higgins-Thompson I^2^ method, and publication bias was determined by Deeks' funnel plot (19, 20). Sensitivity analysis was performed to exclude heterogeneous studies. We performed all the statistical analyses using the STATA, version 12.0 (Stata Corporation, College Station, TX).

## Results

### Literature Search and Description of the Included Studies

**Figure 1:**
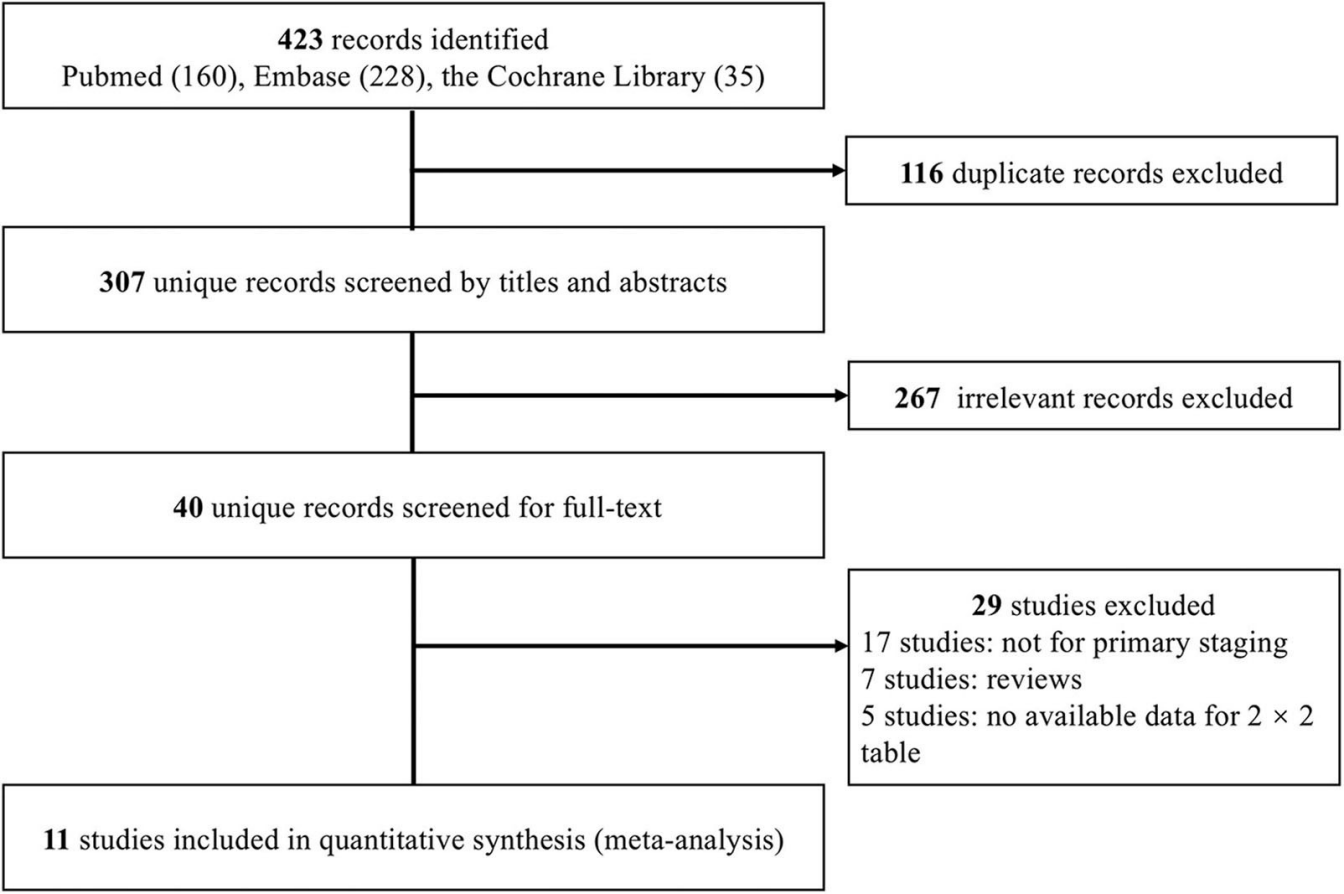
Flow chart of study selection.

We initially identified 423 records. Following the removal of duplicates, 307 records were screened by titles and abstracts, and 40 studies were deemed relevant for full-text assessment. Ultimately, 11 studies (21–31) fulfilled the inclusion criteria and were included in the meta-analysis (Figure 1).

Overall, 11 studies comprising 904 patients who underwent ^68^Ga-PSMA PET/CT before RP with PLND were included. All studies recruited patients with confirmed PCa of intermediate/high risk, and three studies (25, 26, 31) additionally applied nomogram tools (9, 32) to identify patients of high risk of lymph node metastases. All studies set ^68^Ga-PSMA PET/CT imaging as an index test and defined a positive test with increased ^68^Ga-PSMA uptake above background. Of note, one study combined PET information with MRI in a proportion of recruited participants (28). And two studies (24, 25) quantified maximum standardized uptake value (SUV_max_) for positive lymph nodes. The histopathological results from extended PLND (ePLND) were regarded as reference standard in five studies (22, 23, 25, 26, 30), and those from PLND were used as the reference standard in the other six studies. Patients in the studies harbored ages mostly ranging from 60 to 70, while PSA values varied in different studies. Basic information was summarized in Table 1.

**Table 1:** Characteristics of included studies aiming at investigating role of ^68^Ga-PSMA PET/CT for primary lymph node staging in patients with PCa.

**References**	**Country**	**Study design**	**Recruiting period**	**Inclusion criteria**	**CT technique**	**Uptake time (min)**	**Positive PET/CT**	**Reference standard**	**Age (median, range)**	**PSA (median, range)**	**Patient characteristics**	**Tumor stage (*n*, %)**	**Tumor grade (*n*, %)**	**Long axis diameter (mm)**
Klingenberg et al. (31)	Denmark	Retrospective, single center	From April 2016 and March 2019	Biopsy confirmed PCa of high risk, before RP with PLND	CT	~60	Increased PSMA uptake (experienced specialists)	PLND	70.4 (45.2–87.2)	NA	High risk (D'Amico classification) ^g^	NA	NA	NA
Yaxley et al. (21)	Australia	Retrospective, single center	From July 2014 to September 2017	Biopsy confirmed PCa of high/intermediate risk, before RP with PLND	CECT	45–60 (minimum)	Moderate/intense PSMA uptake (experienced radiologists)	PLND	68 (44–80)	7.6 (1.5–51)	Intermediate risk (85) and high risk (123)	NA	Median GS: 4 + 5	4.8 (0.2–40)
van Leeuwen et al. (30)	Australia	Retrospective, multi-center	From February 2015 to October 2017	Biopsy-proven PCa of intermediate/high risk, before RP with ePLND	CECT	45/60	NA (experienced radiologists)	ePLND	NA	9.4	Intermediate risk (30) and high risk (110)	≤ T2 (45, 32.1%) vs. >T2 (95, 67.9%); N0 (89, 63.6%) vs. ≥N1 (51, 36.4%)	GS<8 (46, 32.9%) vs. GS≥8 (98, 67.1%)	NA
Berger et al. (29)	Australia	Retrospective	From February 2015 to January 2017	Biopsy-proven PCa before RP and PLND	CT	Over 60	NA (imaging specialist)	PLND	64.9 ± 5.6	10.6 ± 6.8	Extent of PLND based on patient scenario (median 12 LNs,)	≤ T2 (23, 46%) vs. >T2 (27, 54%)	GS<8 (34, 68%) vs. GS≥8 (16, 32%)	NA
Gupta et al. (22)	India	Retrospective, single center	From December 2014 to December 2015	Confirmed PCa with high risk, before RP with ePLND	CT	~60	Increased PSMA uptake (two physician)	ePLND	61 (46–76)	24.3 (8.7–200.6)	High risk (PSA >20 μg/L, GS 8 or more, and T3 or more)	NA	GS<8 (3, 25%) vs. GS≥8 (9, 75%)	NA
Obek et al. (23)	Turkey	Retrospective, single center	From July 2014 to October 2015	Confirmed PCa of high/very high risk, negative bone scan, before RP, and ePLND	CT	45–60	Visual assessment (two specialists)	ePLND	64 ± 6.0^a^	26.5 ± 21.4^a^	High risk (44) to very high risk (7); Open RP (40), robot assisted RP (11)	≤ T2 (28, 54.9%) vs. >T2 (23, 45.1%); N0 (36, 70.6%) vs. ≥N1 (15, 29.4%)	GS<8 (24, 47.1%) vs. GS≥8 (27, 52.9%)	Detected by PET/CT: 11(5–30) Missed: 4 (0.2–8)
Zhang et al. (24)	China	Retrospective, single center	From March 2017 to July 2017	Biopsy confirmed PCa of high/intermediate risk, before RP with PLND	Non-CECT	60	Increase in tracer activity; qualified SUV_max_ (three physicians)	Bilateral meticulous template PLND^d^	69 (55–82)	37.25 (7.2–348)	Intermediate risk (17) and high risk (25); Robot assisted laparoscopic RP (42)	≤ T2 (11, 26.2%) vs. >T2 (31, 73.8%); N0 (27, 64.3%) vs. ≥N1 (15, 35.7%)	GS<8 (18, 42.9%) vs. GS≥8 (24, 57.1%)	13 (7-31)
van Leeuwen et al. (25)	Australia	Prospective, single center	From April to October 2015	Biopsy confirmed PCa of high/intermediate risk, high risk of LNMs (> 5%)^f^, before RP with ePLND	Non-CECT	60	Visually and quantitatively^e^; semi-quantitatively analyzed by SUV_max_ (two physicians)	ePLND	65 (60–71)^b^	8.1 (5.2–10.1)^b^	Intermediate risk (3), high risk (27); Robotic assistant RP (30)	≤ T2 (9, 30%) vs. >T2 (21, 70%); N0 (19, 63.3%) vs. ≥N1 (11, 36.7%)	GS<8 (7, 23.3%) vs. GS≥8 (23, 76.7%)	Detected by PET/CT: 4.73 ± 1.45 Missed: 2.73 ± 1.29
Budaus et al. (26)	Germany	Retrospective, single center	From June 2014 to March 2015	Confirmed PCa, high risk of LNMs, before RP with ePLND	NA	NA	NA (five imaging centers)	ePLND	63 (44–75)	8.8 (1.4–376)	High risk of LNMs (>20%)^g^	≤ T2 (11, 36.7%) vs. >T2 (19, 63.3%); N0 (18, 60%) vs. ≥N1 (12, 40%)	GS<8 (9, 30%) vs. GS≥8 (21, 70%)	Detected by PET/CT: 13.6 (4–20) Missed 4.3 (1–10.8)
Herlemann et al. (27)	Germany	Retrospective, single center	From January 2014 to August 2015	Confirmed PCa of high/intermediate risk, before RP with PLND	CECT	60	Increased uptake above background	PLND	70.5 (59–80)^c^	56 (3.3–363)^c^	Intermediate risk (4), high risk (16)	≤ T2 (3, 15%) vs. >T2 (17, 85%); N0 (10, 50%) vs. ≥N1 (10, 50%)	GS<8 (10, 50%) vs. GS≥8 (10, 50%)	NA
Maurer et al. (28)	Germany	Retrospective, single center	From December 2012 to November 2014	Confirmed PCa of high/intermediate risk, before RP and PLND	CECT	Mean 59.8 (range 36–165)	Increased uptake (one physician)	Standardized template PLND	66 (45–84)	11.6 (0.57–244)	Intermediate risk (42), high risk (88)	≤ T2 (56, 43.1%) vs. >T2 (74, 56.9%); N0 (89, 68.5%) vs. ≥N1 (41, 31.5%)	NA	NA

a mean ± SD;

b median (IQR);

c mean (range);

d Dissection of all nodes surrounding the common iliac, external iliac, and internal iliac vessels, in the obturator fossa, and in the presacral region, para-aortic and pararectal nodes were removed only if positive sentinel LNs were found;

e Visual analysis included a four-point certainty scoring scale (definitely negative, equivocal probably negative, equivocal probably positive, definitely positive);

f on basis of updated Briganti nomogram;

g *on basis of Briganti's nomogram*.

*CT, computed tomography; PET/CT, Positron Emission Tomography/Computerized Tomography; PSA, prostate specific antigen; RP, radical prostatectomy; PCa, prostate cancer; CECT, contrast enhanced CT; PSMA, prostate-specific membrane antigen; PLND, pelvic lymph node dissection; ePLND, extended PLND; GS, Gleason score; LNMs, lymph node metastases; SUV_max_, maximum standardized uptake value; NA, not available*.

### Diagnostic Accuracy of ^68^Ga-PSMA PET/CT for Preoperative Lymph Node Staging: Per-Patient Analysis

**Figure 2:**
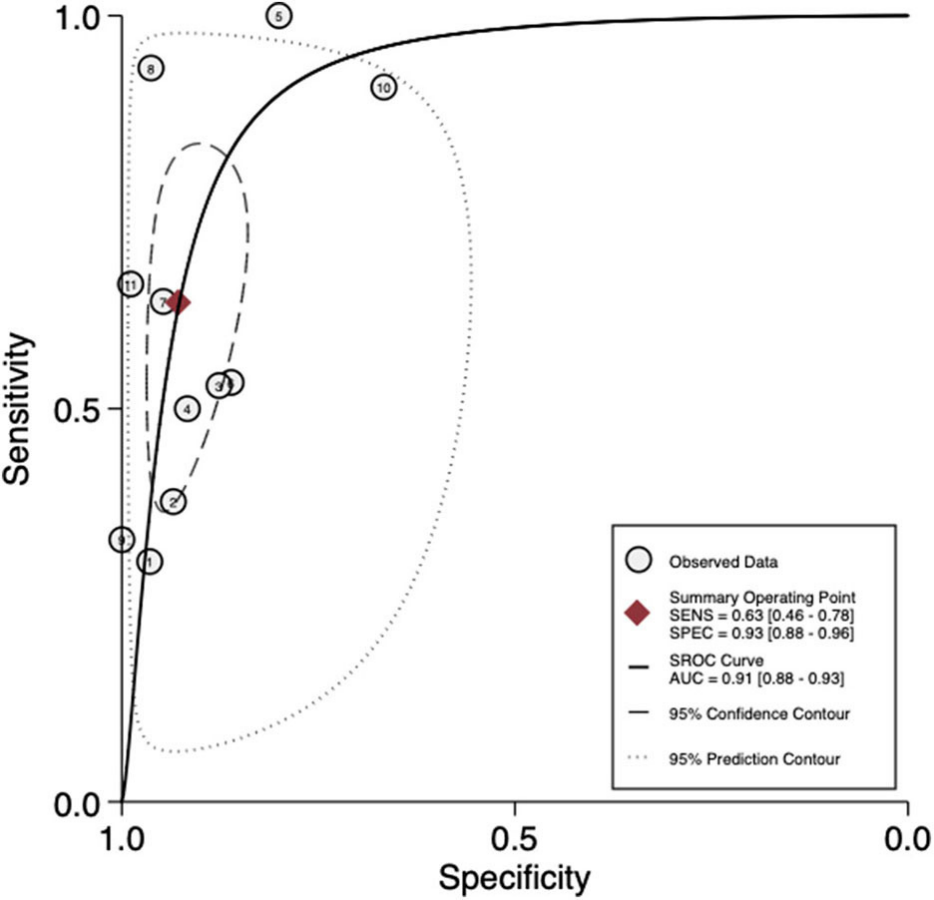
SROC curves for the diagnostic accuracy of ^68^Ga-PSMA PET/CT on a per-patient analysis. SROC curves: summary receiver operating characteristic curves.

For per-patient analysis, 11 studies (21–31) with 904 patients were assessed (Table 2). Of the patients, 16.3% (147/904) and 23.7% (214/904) were diagnosed as positive by ^68^Ga-PSMA PET/CT test and histology of PLND, respectively. The sensitivity and specificity for PET/CT in primary staging ranged from 0.31 to 1.00 and from 0.67 to 1.00, reaching a pooled sensitivity of 0.63 (95% CI: 0.46–0.78), specificity of 0.93 (95% CI: 0.88–0.96), PPV of 0.79 (95% CI: 0.66–0.88), and NPV of 0.84 (95% CI: 0.79–0.89; Table 2). The PLR was 8.7 (95% CI: 5.2–14.5), the NLR was 0.39 (95% CI: 0.25–0.62), and the DOR was 22 (95% CI: 10–47). An overall accuracy was revealed by the SROC curve with AUC of 0.91 (95% CI: 0.88–0.93; Figure 2).

**Table 2:** Summary of studies for ^68^Ga-PSMA PET/CT in primary lymph node staging for PCa patients based on per-patient analysis.

**References**	**No of patients**	**Sensitivity, % (95% CI)**	**Specificity, % (95% CI)**	**PPV, % (95% CI)**	**NPV, % (95% CI)**
**PER-PATIENT ANALYSIS**					
Klingenberg et al. (31)	177	31 (16–48)	96 (92–99)	69 (41–89)	85 (79–90)
Yaxley et al. (21)	208	38 (25–52)	93 (88–97)	68 (49–83)	81 (74–86)
van Leeuwen et al. (30)	140	53 (38–67)	88 (79–94)	71 (54–85)	76 (67–84)
Berger et al. (29)	50	50 (1–99)	92 (80–98)	20 (1–72)	98 (88–100)
Gupta et al. (22)	12	100 (59–100)	80 (28–99)	88 (47–100)	100 (40–100)
Obek et al. (23)	51	53 (27–79)	86 (71–95)	62 (32–86)	82 (66–92)
Zhang et al. (24)	42	93 (68–100)	96 (81–100)	93 (58–100)	96 (81–100)
van Leeuwen et al. (25)	30	64 (31–89)	95 (74–100)	88 (47–100)	82 (60–95)
Budaus et al. (26)	30	33 (10–65)	100 (81–100)	100 (40–100)	69 (48–86)
Herlemann et al. (27)	34^*^	91 (71–99)	67 (35–90)	83 (63–95)	80 (44–97)
Maurer et al. (28)	130	66 (49–80)	99 (94–100)	96 (82–100)	86 (78–92)
**POOLED ANALYSIS**					
	904	63 (46–78)	93 (88–96)	79 (66–88)	84 (79–89)
Pooled PLR	8.7 (5.2–14.5)				
Pooled NLR	0.39 (0.25–0.62)				
Pooled DOR	22 (10–47)				

* *mixed participants; PSMA, prostate-specific membrane antigen; PET/CT, Positron Emission Tomography/Computerized Tomography; PCa, prostate cancer; PLR, positive likelihood ratio; NLR, negative likelihood ratio; DOR, diagnostic odds ratio*.

### Diagnostic Accuracy of ^68^Ga-PSMA PET/CT for Preoperative Lymph Node Staging: Per-Node Analysis

**Figure 3:**
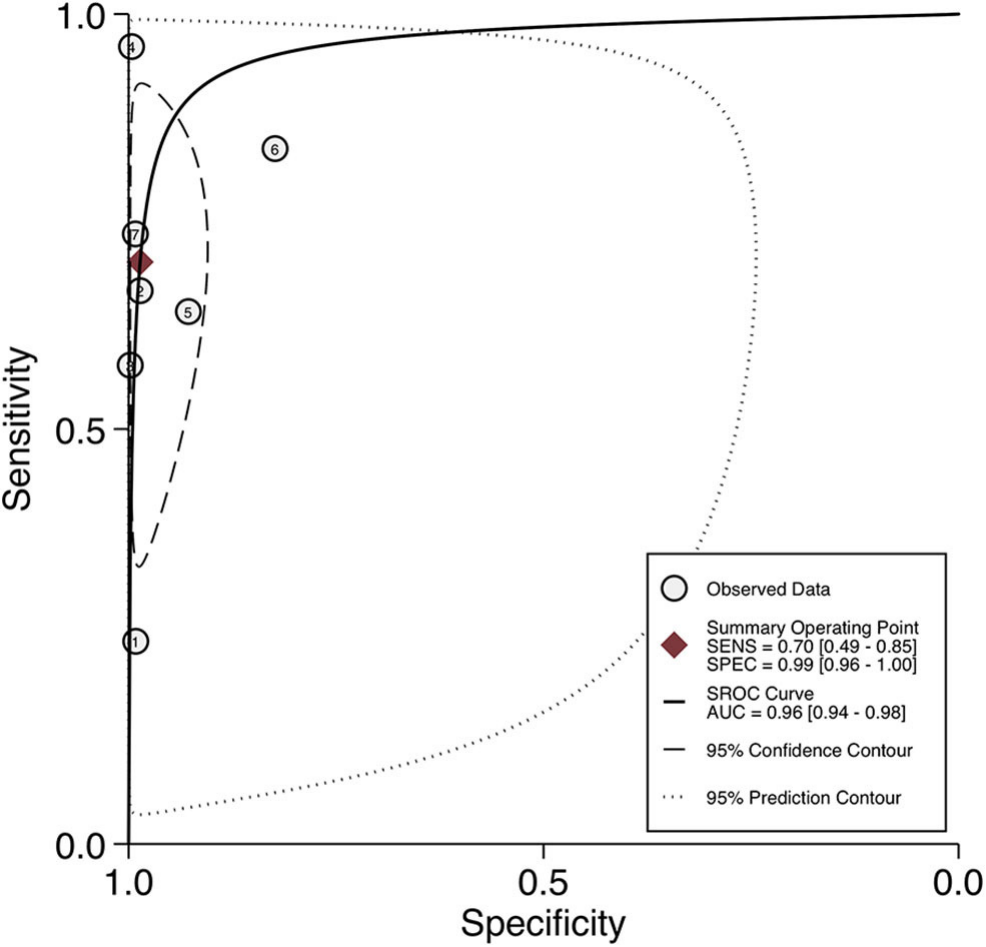
SROC curves for the diagnostic accuracy of ^68^Ga-PSMA PET/CT on a per-node analysis. SROC curves: summary receiver operating characteristic curves.

For per-node analysis, seven studies (21, 22, 24–28) comprising 5,773 lymph nodes were dissected by performing PLND (Table 3). Of the lymph nodes, 6.2% (356/5,773) and 8.4% (483/5,773) were diagnosed as positive by ^68^Ga-PET/CT and histology of PLND, respectively. The sensitivity and specificity for PET/CT in primary staging ranged from 0.24 to 0.96 and from 0.82 to 1.00, reaching a pooled sensitivity of 0.70 (95% CI: 0.49–0.85), specificity of 0.99 (95% CI: 0.96–1.00), PPV of 0.85 (95% CI: 0.69–0.94), and NPV of 0.97 (0.93–0.98; Table 3). The PLR was 50.7 (95% CI: 15.9–162.1), the NLR was 0.30 (95% CI: 0.16–0.56), and the DOR was 167 (95% CI: 40–695). An overall accuracy was revealed by the SROC curve with AUC of 0.96 (95% CI: 0.94–0.98; Figure 3).

**Table 3:** Summary of studies for ^68^Ga-PSMA PET/CT in primary lymph node staging for PCa patients based on per-node analysis.

**References**	**No of lymph nodes**	**Sensitivity, % (95% CI)**	**Specificity, % (95% CI)**	**PPV, % (95% CI)**	**NPV, % (95% CI)**
**PER-NODE ANALYSIS**					
Yaxley et al. (21)	2,960	24 (18–32)	99 (99–99)	64 (51–75)	96 (95–96)
Gupta et al. (22)	243	67 (46–83)	99 (96–100)	86 (64–97)	96 (92–98)
Zhang et al. (24)	621	96 (87–100)	100 (99–100)	96 (87–100)	100 (99–100)
van Leeuwen et al. (25)	536	58 (37–77)	100 (99–100)	94 (70–100)	98 (96–99)
Budaus et al. (26)	608	64 (50–77)	93 (90–95)	46 (34–58)	96 (94–98)
Herlemann et al. (27)	71^*^	84 (68–94)	82 (65–93)	84 (68–94)	82 (65–93)
Maurer et al. (28)	734	74 (65–81)	99 (98–100)	95 (88–98)	95 (93–97)
**POOLED ANALYSIS**					
	5,773	70 (49–85)	99 (96–100)	85 (69–94)	97 (93–98)
Pooled PLR	50.7 (15.9–162.1)				
Pooled NLR	0.30 (0.16–0.56)				
Pooled DOR	167 (40–695)				

* *mixed participants; PSMA, prostate-specific membrane antigen; PET/CT, Positron Emission Tomography/Computerized Tomography; PCa, prostate cancer; PLR, positive likelihood ratio; NLR, negative likelihood ratio; DOR, diagnostic odds ratio*.

### Quality Assessment of the Included Studies

**Figure 4:**
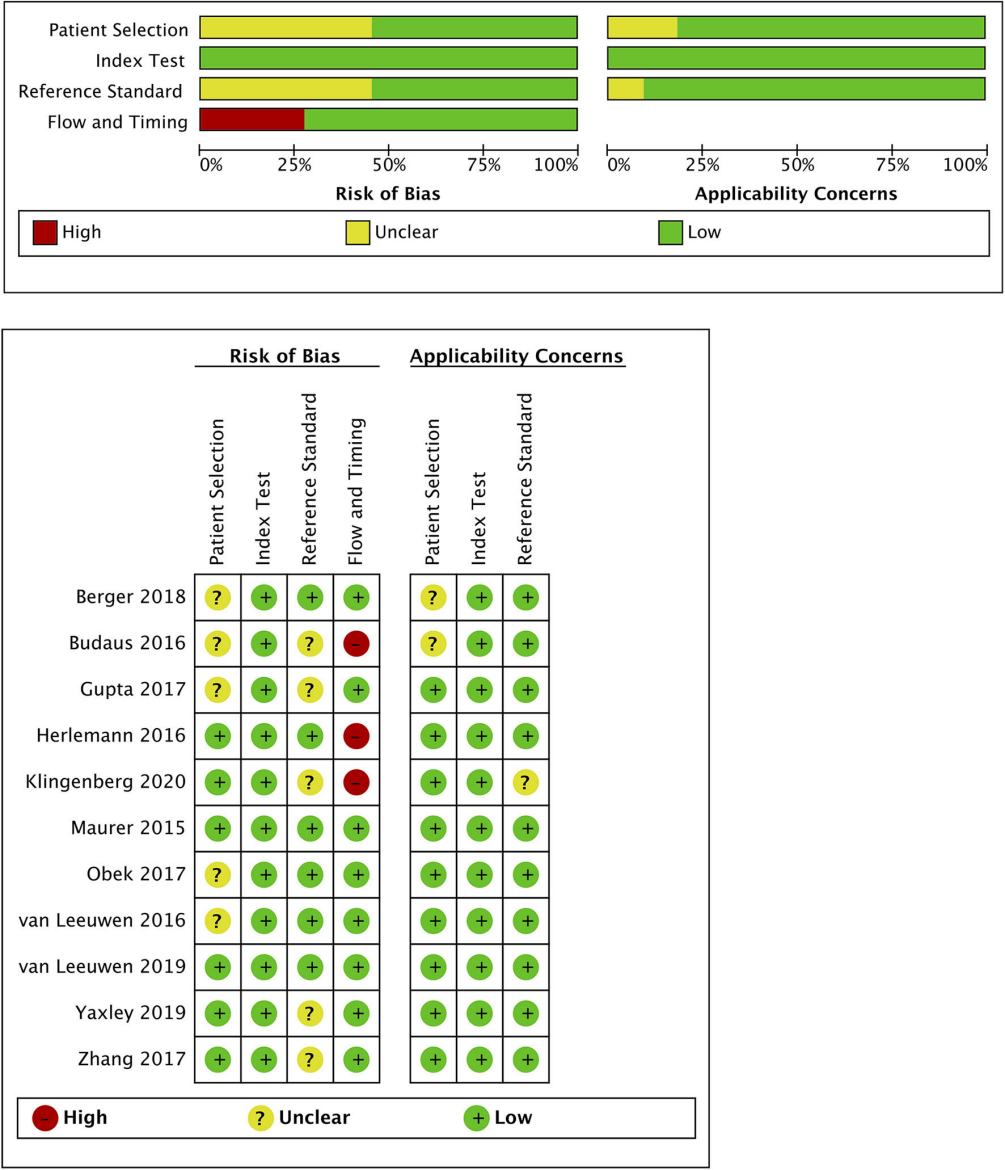
Risk of bias and applicability concerns graph for the included studies.

**Figure 5:**
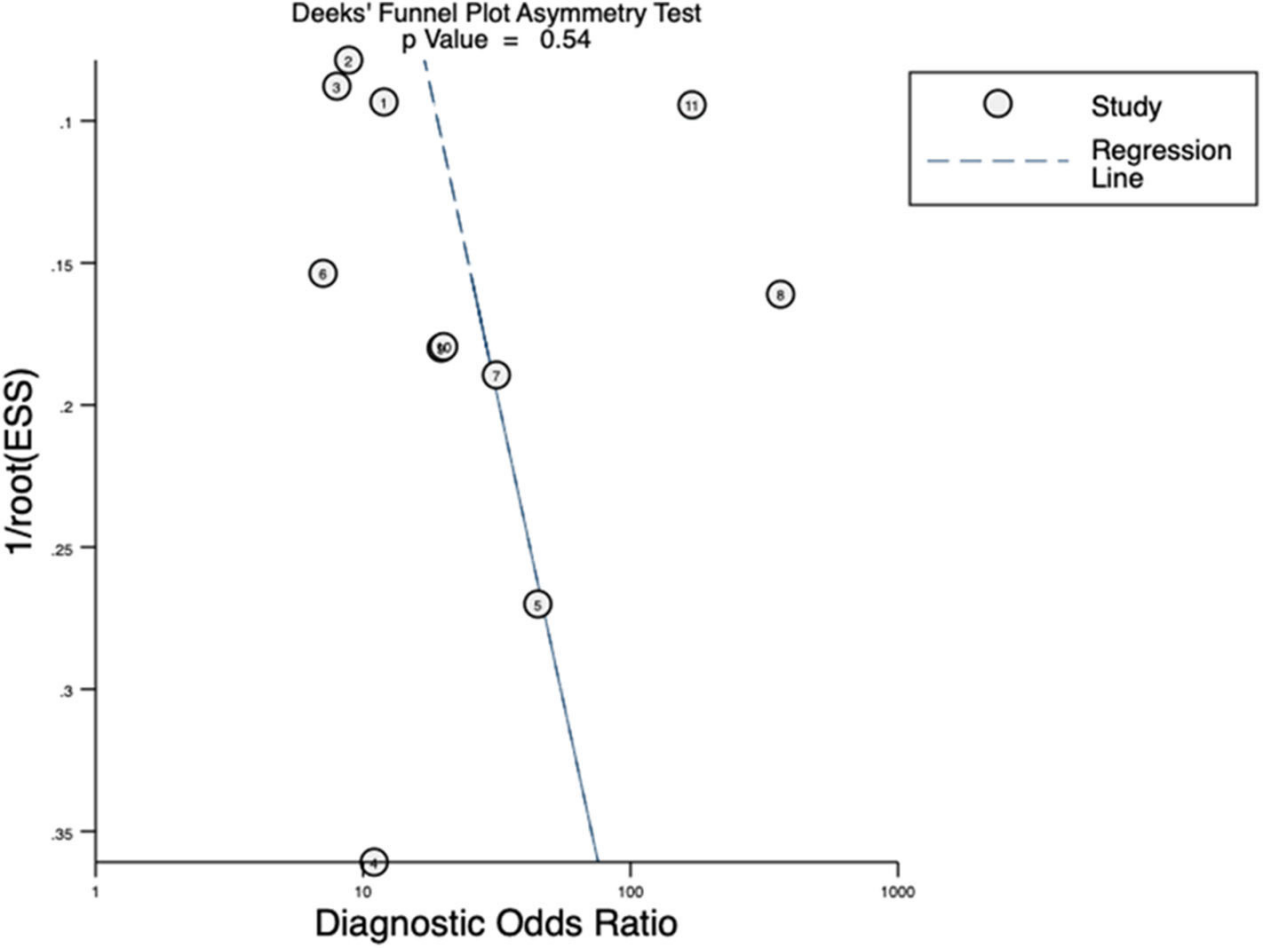
Deeks' funnel plots of publication bias.

Quality assessment of individual studies was summarized in Figure 4. In the patient selection domain, three studies (22, 26, 29) were rated as unclear risk for no specific information (consecutive/random) on patient recruitment. As to the reference standard, four studies (21, 22, 24, 26) were rated as unclear risk because we could not assess whether pathologists were blinded to PET/CT results. In the flow and timing, three studies were considered to be at high risk. In Budaus's study, they initially identified 58 patients; however, their final analyses were restricted to the homogenous cohort with 30 patients (26). And in Herlemann's study, the authors included 14 patients receiving secondary PLND after primary treatment failure, and their final analysis was based on the mixed group (27). And Klingenberg et al. only recruited those patients (177/691) who received PLND for final analysis (31). No publication bias was identified by Deek's funnel plot asymmetry test (*p* = 0.54; Figure 5).

## Discussion

Our study revealed that patients without lymph node metastatic status can rarely be misdiagnosed by PSMA PET/CT. However, the sensitivity of <70% may be not strong enough to forgo additional lymph node staging by PLND.

It is generally accepted that PLND provides important information for staging and prognosis for intermediate/high risk patients of PCa (5). Recently, Abdollah et al. have identified specific categories of pN1 patients (using the PLND information) who would benefit from adjuvant therapy after RP (33, 34). However, PLND itself lacked an definitely therapeutic effect based on current literature (7). Moreover, PLND is associated with worse intraoperative and perioperative outcomes in terms of longer operating time, more blood loss, as well as increased lymphocele/lymphedema rates and venous thromboembolism rates leading to longer hospital stay (6). All these factors raised concern for its routine use in clinical practice. Multiple preoperative nomogram tools have been identified to figure out optimal candidates benefitting from such expensive and invasive procedures (8, 9). However, strict cut-off values of these predictive tools which were originated from various patient selections may not be suitable and optimal for one specific individual, and may put a non-negligible proportion of patients (nearly 12%) under risk of LNI without adjuvant therapy (11).

During the last decades, some more specific as well as non-invasive staging tools for individuals have been investigated. And the conventional imaging procedures used for assessing nodal staging first came to CT and MRI. Hovels et al. performed a meta-analysis on the basis of 24 studies in 2008 to reveal their roles in primary staging using histological evaluation as the gold standard (12). Their results indicated the pooled sensitivity of 0.42 (95% CI 0.26–0.56)/0.39 (95% CI 0.22–0.56) and pooled specificity of 0.82 (95% CI 0.8–0.83)/0.82 (95% CI 0.79–0.83) for CT and MRI, respectively. No significant difference was observed between these two tests. However, both tests were far too intensive in their ability to detect nodal malignancy which can be attributed to using a size criterion of >8 millimeters (morphology) as malignancy (35). In fact, nearly 45% of metastatic lymph nodes are <4 millimeters in diameter which is below the spatial resolution of CT/MRI (36). Moreovenlargement in non-metastatic lymph nodes can mimic a malignant lesion, such as inflammation status or in elderly patients (37). While combining the morphological imaging and functional imaging together, Evangelista et al. performed a systematic review in 2013 and evaluated the diagnostic abilities of ^18^F-choline/11C-choline PET/CT for assessing the involvement of lymph nodes before RP in PCa patients (13). Their meta-analysis included 10 studies with 441 patients. Though with the pooled specificity of 0.95 (95% CI 0.92–0.97), the relatively unsatisfactory sensitivity (0.49, 95% CI 0.40–0.58) limited their routine utility regarding lymph node involvement detection.

To our knowledge, the first meta-analysis investigating the diagnostic accuracy in primary staging of PCa for ^68^Ga-PSMA PET/CT was conducted by von Eyben et al. in 2016 (38). They included four available studies revealing a pooled sensitivity of 0.61 (95% CI 0.47–0.72) and specificity of 0.97 (95% CI 0.85–0.99) in patient-based analysis. And the pooled sensitivity and specificity in node-based analysis were 0.70 (95% CI 0.53–0.83) and 0.84 (95% CI 0.24–0.99), respectively. Thereafter, Corfield et al. performed a critical review, recruiting nearly the same study cohorts, and summarized the role of ^68^Ga-PSMA PET/CT for primary staging of high-risk PCa (39). Their result revealed PSMA PET/CT appeared to outperform traditional imaging modalities but without performing quantitative synthesis. Perera's group also have tried to synthesize the role of ^68^Ga-PSMA PET in patients of advanced PCa; however, they mainly focused on assessing predictors of positive tests for patients with biochemical recurrence (40). And further updated work reported the predictive ability of PSMA-PET/CT imaging for primary staging. Of note, failure of including currently all available evidence may cause potential bias (41, 42).

To provide more concise and updated evidence on the role of ^68^Ga-PSMA PET/CT for predicting lymph node metastases before definitive treatment, we conducted a thorough literature research and included currently all available studies. Using histological status of resected lymph nodes as the reference standard, our study found the pooled specificity can reach above 90% both in per-patient analysis and in per-node analysis, which indicated that patients without lymph node metastatic status can rarely be misdiagnosed by ^68^Ga-PSMA PET/CT. The relatively low misdiagnosis rate can be further supported with the superior PPV and NPV (above 80%). However, PSMA PET/CT failed to reach high diagnostic evidence criteria defined by Jaeschke et al. (PLR of 5–10 and NLR of 0.1–0.2) (43), mainly due to its relatively low sensitivity. With a sensitivity of <70%, if completely on the basis of ^68^Ga-PSMA PET/CT test, more than 30 out of 100 truly positive patients with metastatic lymph nodes would be missed and thereafter be under additional risk of progression without adjuvant therapy. However, these values were superior to those for traditional imaging approaches. The staging performance of ^68^Ga-PET imaging co-registered with MRI was currently less investigated. Grubmuller et al. prospectively recruited 80 patients who received preoperative PSMA-PET/mpMRI followed by RP and ePLND and reported a sensitivity of 68.8% and a specificity of 100% for the prediction of lymph node metastasis on patient base (44). Thalgott's retrospective study recruiting 73 patients of high-risk prostate cancer has reported comparable results (sensitivity: 60%; specificity 100%) (45).

Our study has several limitations. Firstly, the majority data was derived from retrospective, single-institutional studies with small sample sizes. However, through a careful and thorough literature search, our study provided the most up-to-date evidence on basis of per-patient and per-node analysis. Secondly, there was considerable heterogeneity in our pooled analysis. We assumed some confounders may have impact on the different diagnostic accuracy across included cohorts. For example, the sensitivity and specificity in Zhang's study (24) were obviously higher than those in Yaxley's study (21), which can be partly owing to different PSA levels between their cohorts (median 37.25 vs. 7.7 ng/ml). Higher diagnostic accuracy was observed in Herlemann's study which included a portion of patients receiving salvage PLND (27). Varied diameters of lymph nodes caused by different inclusion PLND criteria (e.g., using nomogram tool or not) may also contribute to the heterogeneity (24–26). Moreover, the application of PLND or ePLND, the different sample size (22), study quality, and experiences of radiologists/urologists and pathologists may also have impacts. Nevertheless, they are regarded as common pitfalls for real-world clinical practice. Thirdly, as a review, we cannot correlate the positive lymph nodes with the preoperative PSA level and the Gleason score. In combination with such parameters may improve sensitivity and specificity of PSMA PET/CT. We also cannot evaluate the role of SUVmax value due to limited data available. Finally, issues about the additional cost, clinical feasibility, and potential benefit should also be considered and balanced for individuals in clinical practice, however, which are out of the scope of our study.

In conclusion, we synthesized currently available evidence to evaluate the role of ^68^Ga-PSMA PET/CT for preoperative lymph nodes staging in patients with intermediate/high risk PCa. Our study found patients without lymph node metastatic status can rarely be misdiagnosed by PSMA PET/CT. However, the relatively low sensitivity of <70%, though superior to that for traditional imaging approaches, is not strong enough to forgo lymph node staging by PLND. Future studies should focus on the evaluation of lymph node involvement in a stricter patient selection, such as those with a very high risk of lymph node dissemination or those specific patients involved in controversial status of lymph node metastasis based on current preoperative risk estimation nomograms.

## Take-Home Message

1. ^68^Ga-PSMA PET/CT can provide more accurate staging information of lymph nodes (Sen 0.63, Spe 0.93) for intermediate/high risk PCa compared with traditional imaging approaches (CT: Sen 0.42, Spe 0.39; MRI: Sen 0.39, Spe 0.82; ^18^F-choline/^11^C-choline PET/CT: Sen 0.49, Spe 0.95).
2. Patients without lymph node metastatic status can rarely be misdiagnosed by ^68^Ga-PSMA PET/CT (Spe: 0.93, 95% CI 0.88–0.96). However, the pooled sensitivity (0.63, 95% CI 0.46–0.78) indicated more than 30 out of 100 truly positive patients with metastatic lymph nodes would be missed and thereafter be under additional risk of progression without adjuvant therapy.

## Data Availability Statement

All datasets presented in this study are included in the article/supplementary material.

## Author Contributions

XT, CZ, and ZL: protocol development, data collection or management, data analysis, and manuscript drafting. GS and XW: data management, manuscript review, and revision. LN: data management, manuscript review, and revision. TC: data collection and management. HX and YB: data collection or management and data analysis. LY and QW: study concept and design, manuscript review, and supervision. All authors contributed to the article and approved the submitted version.

## Conflict of Interest

The authors declare that the research was conducted in the absence of any commercial or financial relationships that could be construed as a potential conflict of interest.

## Abbreviations

PET/CT: positron emission tomography/computerized tomography
PCa: prostate cancer
PLND: pelvic lymph node dissection
RP: radical prostatectomy
PPV: positive predictive value
NPV: negative predictive value
SROC: summary receiver operating characteristic
AUC: area under the curve
LVI: lymph node invasion
CT: computed tomography
MRI: magnetic resonance imaging
PSMA: prostate-specific membrane antigen
PRISMA: Preferred Reporting Items for Systematic Review and Meta-analysis
PSA: prostate specific antigen
QUADAS: the Quality Assessment Tool for Diagnosis Accuracy Studies
PLR: positive likelihood ratio
NLR: negative likelihood ratio
DOR: diagnostic odds ratio
ePLND: extended pelvic lymph node dissection.
